# Supplementary material for: DMRforPairs: identifying Differentially Methylated Regions between unique samples using array based methylation profiles
Source: BMC Bioinformatics. 2014 May 15;15:141. doi: 10.1186/1471-2105-15-141 (PMC4046028; doi:10.1186/1471-2105-15-141)
Supplement: Additional file 2 — DMRforPairs output for the comparison of A431-MCF7 and NA17018-NA17105. Please start from the HTML files in each folder. Available via the BMC Bioinformatics website. [file 1471-2105-15-141-S2.zip › 1394847754114233_MOESM2_ESM/A431_MCF7/figures/23723.pdf]

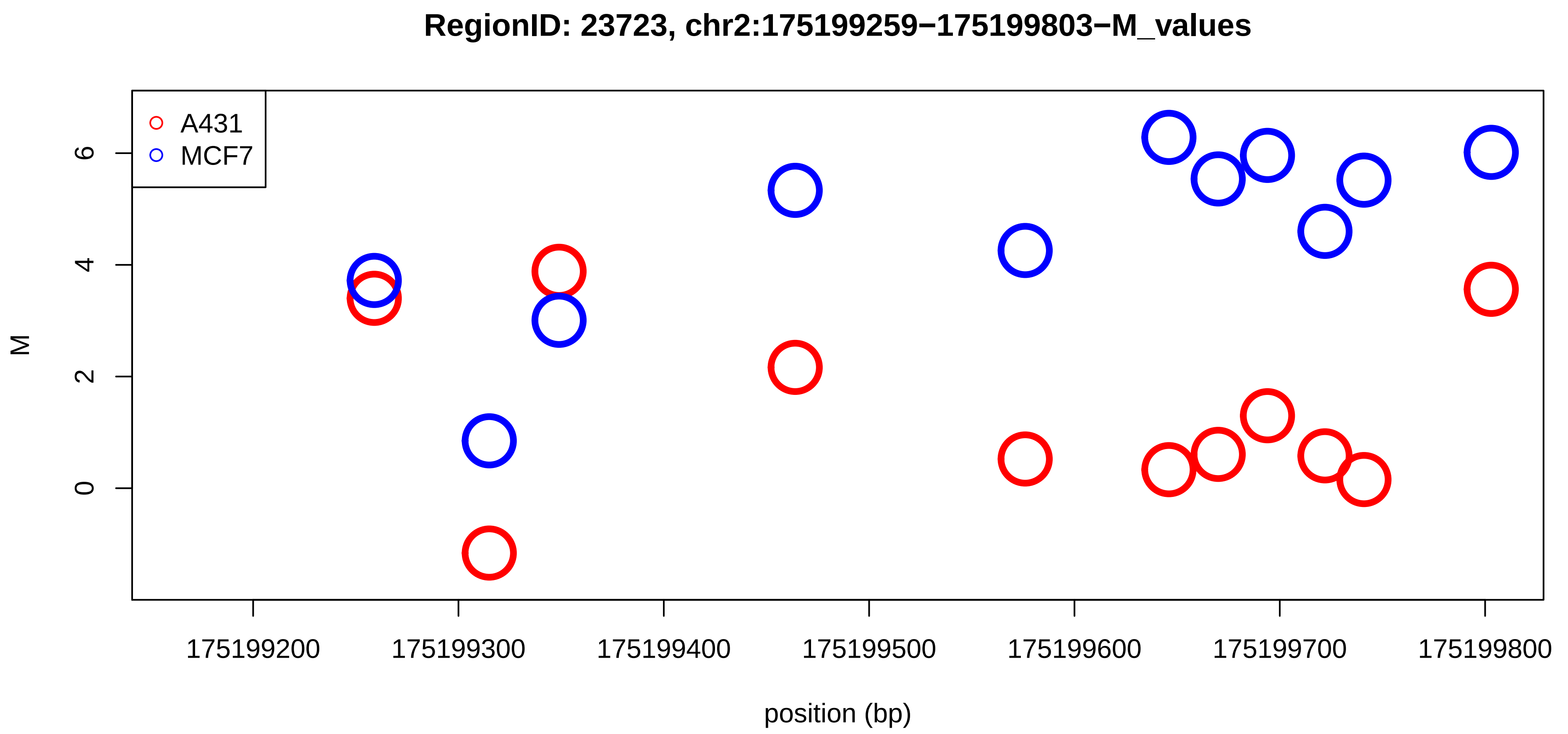
RegionID: 23723, chr2:175199259−175199803−M_values
A431
MCF7
M
0
2
4
6
175199200
175199300
175199400
175199500
175199600
175199700
175199800
position (bp)

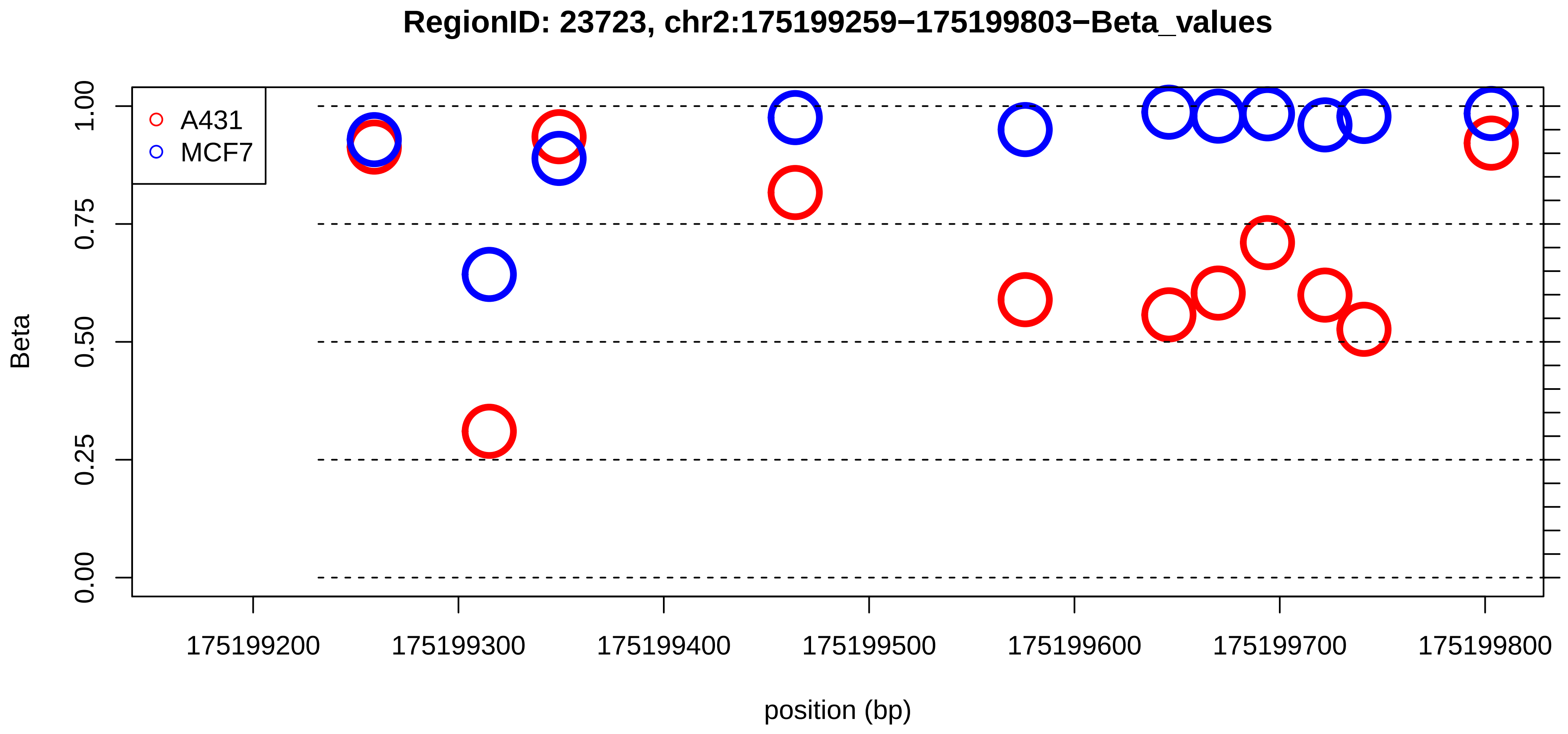
RegionID: 23723, chr2:175199259−175199803−Beta_values
A431
MCF7
Beta
0.00
0.25
0.50
0.75
1.00
175199200
175199300
175199400
175199500
175199600
175199700
175199800
position (bp)

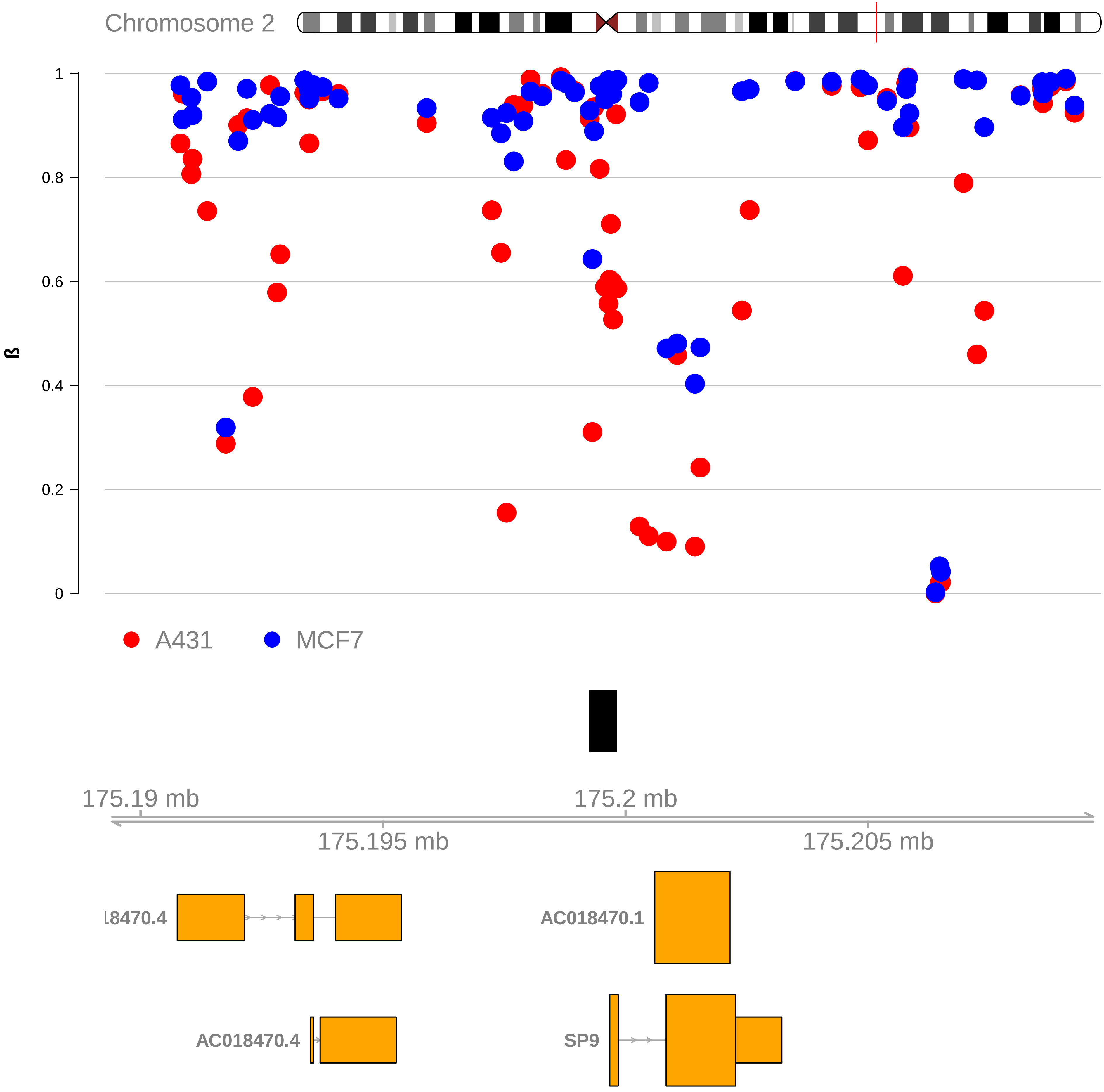

Chromosome 2
1
0.8
0.6
0.4
0.2
0
ß
A431
MCF7
175.19 mb
175.2 mb
175.195 mb
175.205 mb
18470.4
AC018470.1
AC018470.4
SP9
